# Poor school performance and gambling among adolescents: Can the association be moderated by conditions in school?

**DOI:** 10.1016/j.abrep.2023.100508

**Published:** 2023-07-17

**Authors:** Joakim Wahlström, Gabriella Olsson

**Affiliations:** Department of Public Health Sciences, Stockholm University. Albanovägen 12, 106 91 Stockholm, Sweden

**Keywords:** Gambling, Risk gambling, School performance, Student–teacher ratio, Youth

## Abstract

**Introduction:**

Protective factors of adolescent gambling at the school level and their buffering potential are scarcely explored in prior research. This study aims to examine the protective possibility of low student–teacher ratio on youth gambling, both directly and by moderating the effect of low school performance.

**Methods:**

Data were derived from the 2016 Stockholm school survey, collected among 5,221 grade 11 students (∼17–18 years) in 46 schools, with information on schools’ composition and student–teacher ratio obtained through registers. Gambling and risk gambling were coded as binary variables. School performance was captured by self-reported marks in three core subjects, dichotomised into average/above average and below average, respectively. Student-teacher ratio was used both as a continuous and trichotomised variable. Two-level binary logistic regression analyses were performed.

**Results:**

A below average school performance was associated with gambling and risk gambling but the association with gambling was only statistically significant at the 10%-level in the fully adjusted model. Student-teacher ratio was not directly associated with gambling and risk gambling but moderated the associations between school performance and both gambling and risk gambling, as these relationships were less pronounced in schools with a low student–teacher ratio.

**Conclusions:**

In sum, a low student–teacher ratio may protect students from gambling and risk gambling by buffering against the adverse effects of other risk factors, such as poor school performance. These findings suggest that a higher teacher density in upper secondary schools can be beneficial beyond school matters by positively influencing student behaviour outside of school.

## Highlights

• School performance was associated with gambling and risk gambling.
• Student-teacher ratio (STR) moderated these associations.
• Favourable conditions in school may mitigate risk factors for youth gambling.
• A lower STR can be beneficial for students beyond educational achievements.
• Increasing the number of teachers could be relevant for public health policies.

## 1 Introduction

Despite an age limit of 18 years, gambling among Swedish minors is highly prevalent and about 3–5% of 15–18-year-olds in Sweden are considered to be at-risk or problem gamblers (CAN, 2021, The Public Health Agency of Sweden, 2022, The Social Administration in Stockholm, 2022). Gambling-related problems among youth include impaired mental health, strained relationships, and school difficulties (Dowling et al., 2017). Risk gambling encompasses behaviours such as betting for increasing amounts of money or borrowing money to keep gambling (CAN, 2021). Early onset of problem gambling is associated with an elevated risk of developing gambling problems later in life but may also have short- and long-term social, educational, economic and health-related consequences, both for the individual and for people in their immediate social environment (Riley et al., 2021, Tulloch et al., 2022). Counteracting adolescent gambling is thus a public health issue and identifying conditions that protect youth at risk of gambling is of great importance.

Previous research has highlighted several important risk and protective factors for youth gambling at the individual and family level. It is for instance well established that mental health issues, impulsivity and sensation-seeking personality traits, parental gambling, and sociodemographic characteristics including male gender, poor socioeconomic conditions, living in a single-parent household, and belonging to an ethnic minority are associated with greater risks of adolescent gambling and problem gambling (Calado et al., 2017, Claesdotter-Knutsson et al., 2022, Dickson et al., 2008, Dowling et al., 2017, Lee et al., 2014, McComb and Sabiston, 2010, Shead et al., 2010, Svensson and Sundqvist, 2019). While the research on protective factors is not as exhaustive, prior studies have, for example, found that family and peer support, parental monitoring, family cohesion, and effective coping styles are inversely associated with problematic gambling among adolescents (Dickson et al., 2008, Elgar et al., 2018, McComb and Sabiston, 2010, Shead et al., 2010).

Turning to the school environment, where adolescents spend a substantial amount of their time, factors such as academic achievement, school connectedness, and classmate support have been identified as inversely associated with involvement in gambling (Dickson et al., 2008, Elgar et al., 2018, Shead et al., 2010). Key risk factors include poor school performance and truancy (Dowling et al., 2017, Wahlström et al., 2022). Furthermore, supportive and caring teacher-student relationships may protect against student gambling (Elgar et al., 2018, Räsänen et al., 2016, Wahlström et al., 2022) and could positively impact other aspects of young peoples’ lives as well (Låftman and Modin, 2012, Modin and Östberg, 2009, Quin, 2017, Rudasill et al., 2010). However, teachers encounter significant challenges in providing support to students. High levels of teacher stress, which are reported both in Sweden (Swedish Governmental Official Reports (SGOR)., 2014, SNAE, 2019, SWEA, 2016) and in other countries (Hakanen et al., 2006, OECD., 2020), can impede the development of caring teacher-student relationships and negatively impact student outcomes (Arens and Morin, 2016, Klusmann et al., 2016, Ramberg et al., 2020, Shen et al., 2015). One can hypothesize that by reducing teachers' workload to counteract work stress, students may experience increased teacher support, which can serve as a protective factor against gambling behaviour. Higher teacher density (i.e., more teachers per student) is likely to mitigate teacher stress and has previously been linked to better student outcomes in Sweden (Andersson, 2007, Björklund et al., 2005). A higher teacher density probably leads to increased teacher-student contacts, which in turn may facilitate supportive bonds between teachers and students.

Beyond directly influencing the likelihood of adolescent gambling, protective factors can also alleviate the negative effects of risk factors. Prior research has indicated that studies investigating the moderating effects of protective factors are needed (Dowling et al., 2017). Conditions at the school level may be particularly relevant to examine in this regard, given the potential for schools to intervene effectively. Related research has indicated that school-level conditions can interact with individual and/or family factors, buffering the impact of risk factors on other outcomes than gambling (Crosnoe, 2004, Eklund and Fritzell, 2014, Olsson and Fritzell, 2017, Olsson et al., 2021a, Watson et al., 2021).

### 1.1 The present study

The purpose of this study is to examine the moderating effect of student–teacher ratio, i.e., the opposite of teacher density, on the association between school performance and engagement in gambling and risk gambling. It is assumed that the protective effect of a low student–teacher ratio on (risk) gambling may act in two ways: first, by directly reducing the likelihood of (risk) gambling, measured via its main effects; and second, by moderating the effect of known risk factors, in the case of this study, the impact of low school performance on (risk) gambling. When student–teacher ratio is low (i.e., fewer students per teacher), we hypothesise that the strength of the relationship between low school performance and (risk) gambling is weaker.

## 2 Materials and Methods

### 2.1 Data

The study uses data from the Stockholm School Survey (SSS) with added information from administrative registers. The SSS is conducted biennially among students in grades 9, the last year of compulsory school (aged≈15-16), and 11, the second year of upper secondary school (aged≈17-18), attending schools in Stockholm municipality (The Social Administration in Stockholm, 2022). All schools are offered the opportunity to participate in the survey, with public schools required to do so while private schools may choose to refrain. The survey is distributed in classrooms by teachers, answered anonymously by students through pencil-and-paper, and the completed questionnaires are returned in sealed envelopes. Administrative register information on schools from the Swedish National Agency for Education has been linked to this data. In the current study, data from 6,129 11th grade students (response rate 77.1%) in 58 identified upper secondary schools from the 2016 survey were used. Although almost two-thirds of the schools that participated were private (≈64%) a majority of the students attended public schools (≈58%). Due to lacking information at the school level, 525 students from 12 schools were excluded and after further exclusion due to missing data on any of the student-level variables, the final study sample consisted of 5,221 students in 46 schools. Since the SSS is completed anonymously and no information on personal identification is provided, the Regional Ethical Review Board of Stockholm has decided that these data are not under consideration for ethical approval (ref. no. 2010/241–31/5).

### 2.2 Measures

#### 2.2.1 Outcome variables

*Gambling* was measured by asking students: “Have you bought lottery tickets or gambled for money at any time during the last 12 months?”, with the specification “(Scratch ticket, game show lottery, casino, poker, betting on football, horses or the like, also on the Internet)”. Those who answered “Yes” was categorised as having gambled. This measure has been used in previous studies (Låftman et al., 2020b, Låftman et al., 2020a, Olsson et al., 2021b, Wahlström et al., 2022).

*Risk gambling* was assessed by three questions, posed to those who had gambled the last 12 months: “How many times during the last 12 months have you”: a) “Tried to reduce your gambling?”, b) “Felt restless and irritated if you haven’t been able to gamble”, and c) “Lied about how much you’ve gambled?”. There were three response categories: “Never”, “1–2 times”, and “3 times or more”. Students who answered “1–2 times” or “3 times or more” on at least one of the three items were categorised as risk gamblers. This measure of risk gambling has been used in prior studies (Kaltenegger et al., 2019, Låftman et al., 2020a, Låftman et al., 2020b; Olsson et al., 2021b; Wahlström et al., 2022).

#### 2.2.2 Individual-level variables and covariates

*School performance* was captured by the students’ self-reported marks in Swedish, English, and mathematics. Each mark was given a corresponding number (A = 5, B = 4, C = 3, D = 2, E = 1, F = 0, ‘No mark’=0), and summed to an index ranging from 0 to 15. Missing answers on one of the subjects were replaced by the individual mean of the remaining subjects. The variable was dichotomised into students with low marks (one standard deviation below the mean) and students with average/high marks. Covariates included *gender*, *family structure*, *parental university education*, and *migration background*. Gender was assessed by the question: “Are you a boy or a girl?”. Students who skipped this question were kept in a separate category. Family structure was based on the question: “Which people do you live with?” and a list of possible family members that the respondents could select. Those who marked that they lived with both their mother and father in the same household were contrasted against all other family structures. For parental university education, respondents were asked about their parents’ highest education, separately for mothers and fathers, with the response alternatives: “Compulsory school”, “Upper secondary school”, and “University”. Those with at least one university-educated parent was contrasted against all others. Migration background was based on the question: “How long have you lived in Sweden?”. The respondents were given four response alternatives: “All my life”, “10 years or more”, “5–9 years”, and “<5 years”. The variable was dichotomised and distinguished between respondents who had lived in Sweden at least 10 years and those who had lived in Sweden for<10 years.

#### 2.2.3 School-level variables and covariates

The school-level variables were based on information from the Swedish National Agency for Education. *Student-teacher ratio* captured the number of students per teacher in the school. *Proportion of parents with post-secondary education* was defined as the share of students with at least one parent with tertiary education in the school. *Proportion of students with a foreign background* was defined as the school percentage of students with both parents born abroad.

### 2.3 Statistical methods

Initially, the associations between gambling, risk gambling, and school performance were assessed through crosstabulations and chi-square tests. Then, two-level binary logistic regression Models with gambling and risk gambling as outcome variables were performed using the “melogit” command in Stata, version 17.0. First, we fitted an empty Model. Model 1 added school performance and all individual-level covariates. In Model 2, student–teacher ratio was included as well. In Model 3 the proportion of parents with post-secondary education and the proportion of students with a foreign background was added. Lastly, in Model 4, the interaction term between school performance and student–teacher ratio was included in the analysis. For all Models, the intra class correlation (ICC) is reported, to provide an approximation of the amount of variation that can be attributed to the higher level. Additionally, fully adjusted analyses were performed stratified by categories of the schools’ student–teacher ratio (low, intermediate, and high).

## 3 Results

Descriptive statistics for the individual-level and school-level variables are presented in Table 1. Gambling during the last 12 months was reported by 14.8% of the students and 3.3% were regarded as risk gamblers. As for school performance, 14.8% were categorised as having low marks. Regarding the school-level variables, there were large differences between schools. The average proportion of gamblers in schools were 16.4%, however this figure ranged between 3.4% and 43.6% depending on the school. With regards to risk gambling, the average school had 4.0% risk gamblers and the proportion varied between 0% and 15.4%. The average number of students per teacher (student–teacher ratio) was 16, and varied between 5.1 and 26.7 across schools. Similarly, the variation across schools was wide for the proportion of parents with secondary education (average = 42.2%) and the proportion of students with foreign background (average = 46.3%).

**Table 1:** Descriptive statistics, n = 5,221.

**Variables**				
*Individual level*				
	n	%		
Gambling				
No	4,447	85.2		
Yes	774	14.8		
Risk gambling	174	3.3		
School performance				
Average/above average	4,450	85.2		
Low	771	14.8		
Gender				
Girls	2,717	52.0		
Boys	2,347	45.0		
Missing	157	3.0		
Family structure				
Living with two parents in the same household	3,346	64.1		
Other	1,875	35.9		
Parents’ university education				
No parent	1,724	33.0		
At least one parent	3,497	67.0		
Migration background				
Born in or lived in Sweden ≥ 10 years	4,763	91.2		
Lived in Sweden < 10 years	458	8.8		
*School level*				
	Mean	S.d.	Min	Max
Proportion of students having engaged in gambling	16.4	8.99	3.4	43.6
Proportion of students having engaged in risk gambling	4.0	3.90	0	15.4
Student-teacher ratio	16.0	3.98	5.1	26.7
Proportion of parents with post-secondary education	42.2	23.13	7	86.3
Proportion of students with foreign background	46.3	22.27	6	95.7

In Table 2, the proportion of students that had gambled and risk gambled by school performance, for the full sample and stratified by the schools’ student–teacher ratio, are presented. For these analyses, we trichotomised the student–teacher ratio variable. Chi-square tests were performed to assess differences between groups. In the total sample, gambling was reported by 14.1% of the students with an average or above average school performance. The corresponding figure for those with low school performance was 19.1%. The difference was statistically significant (*p* < 0.001). The analyses stratified by schools’ student–teacher ratio showed that gambling was overall more prevalent among students with poorer school performance compared to those with better school performance. However, the difference between the two categories was more substantial among students attending schools with a high student–teacher ratio compared with students attending schools with intermediate or low student–teacher ratio. The differences between low and average/above-average performing students was statistically significant in schools with an intermediate and high student–teacher ratio. For risk gambling, the pattern was the same but the difference was more pronounced. In the total sample, risk gambling was reported by 2.8% of the students with an average or above average school performance. The corresponding figure for those with a low school performance was 6.6%. The analyses stratified by schools’ student–teacher ratio showed that risk gambling was overall more prevalent among students with poorer school performance compared to those with a better school performance. All differences were statistically significant.

**Table 2:** Proportion of students gambling and risk gambling by school performance. Total sample and stratified by student–teacher ratio, n = 5,221.

	Gambling							
	Total sample (n = 5,221)		Low student–teacher ratio (n = 1,682)		Intermediate student–teacher ratio (n = 1,676)		High student–teacher ratio (n = 1,863)	
School Performance	n	%	n	%	n	%	n	%
Average/above average	627	14.1	176	13.8	214	14.4	237	14.1
Low	147	19.1	70	17.4	37	20.0	40	21.9
χ^2^		12.9***		3.2^+^		4.1*		7.8**
	Risk gambling							
	Total sample (n = 5,221)		Low student–teacher ratio (n = 1,682)		Intermediate student–teacher ratio (n = 1,676)		High student–teacher ratio (n = 1,863)	
School Performance	n	%	n	%	n	%	n	%
Average/above average	123	2.8	48	3.8	33	2.2	42	2.5
Low	51	6.6	27	6.7	10	5.4	14	7.7
χ^2^		30.2***		6.2*		6.7*		15.0***

*Note*. ***p < 0.001 **p < 0.01 *p < 0.05 ^†^*p* < 0.10.

Table 3 displays odds ratios (OR) from two-level binary logistic analyses with student gambling and risk gambling as the outcome variables. With regards to gambling, the empty Model A shows that 8.3% of the total variation in gambling occurred at the school level. In Model 1A the association between gambling and school performance is explored while adjusting for the individual-level covariates. Students with a low school performance were more likely to have gambled compared with students with an average or above average school performance (OR 1.24; *p* < 0.10). Information on the schools’ student–teacher ratio was added in Model 2A but was not associated with gambling (OR 1.00). The inclusion of the other school-level variables in Model 3A did not produce any noteworthy changes in the associations. In Model 4A the interaction between school performance and student–teacher ratio was included in the analysis. The interaction term was associated with gambling (OR 1.07; *p* < 0.05), indicating that the link between low school performance and gambling was stronger in schools with a higher student–teacher ratio. Additionally, boys and individuals who skipped the question regarding their gender were more likely to have engaged in gambling compared to girls.

**Table 3:** Odds ratios from two-level binary logistic regression of gambling and risk gambling, n = 5,221 in 46 schools.

	**Gambling**					**Risk gambling**				
	**Empty Model A**	**Model 1A**	**Model 2A**	**Model 3A**	**Model 4A**	**Empty Model B**	**Model 1B**	**Model 2B**	**Model 3B**	**Model 4B**
		OR	OR	OR	OR		OR	OR	OR	OR
School performance										
Average/above average (ref.)		1.00	1.00	1.00			1.00	1.00	1.00	
Low		1.24^+^	1.24^+^	1.25^+^			1.73**	1.70**	1.73**	
Gender										
Girls (ref.)		1.00	1.00	1.00			1.00	1.00	1.00	
Boys		3.99***	3.99***	3.93***			12.06***	12.01***	11.83***	
Missing		1.95**	1.95**	1.93**			5.60**	5.58**	5.47**	
Family structure										
Living with two parents in the same household (ref.)		1.00	1.00	1.00			1.00	1.00	1.00	
Other		1.18^+^	1.18^+^	1.18^+^			1.33^+^	1.32^+^	1.32^+^	
Parental university education										
At least one parent (ref.)		1.00	1.00	1.00			1.00	1.00	1.00	
No parent		1.13	1.13	1.12			1.51*	1.50*	1.48*	
Migration background										
Born in or lived 10 + years in Sweden (ref.)		1.00	1.00	1.00			1.00	1.00	1.00	
Lived in Sweden<10 years		0.80	0.80	0.82			1.22	1.21	1.27	
*School level*										
Student-teacher ratio			1.00	1.01				0.98	0.99	
Proportion of parents with post-secondary education				0.99**					0.99	
Proportion of students with foreign background				0.99					1.00	
*Cross level interaction^a^*										
School performance*Teacher density					1.07*					1.11*
ICC	8.3%	5.1%	5.1%	4.0%		15.7%	4.3%	4.2%	3.0%	

*Note*. ^a^ Cross level interaction Models include all exposure variables and covariates ***p < 0.001 **p < 0.01 *p < 0.05 ^†^*p* < 0.10.

Turning to risk gambling as the outcome, the empty Model B shows that of the total variation, 15.7% could be attributed to differences between schools. Model 1B reveals that students with a low school performance were more likely to engage in risk gambling compared with students with an average or above average school performance (OR 1.73; *p* < 0.01). Model 2b shows that student–teacher ratio was not associated with risk gambling (OR 0.98). Again, including the covariates at the school level in Model 3B did not affect the associations of interest. Finally, in Model 4B, the interaction term between school performance and student–teacher ratio was associated with risk gambling (OR 1.11; *p* < 0.05), indicating that the association between low school performance and gambling was stronger in schools with a higher student–teacher ratio. Moreover, an elevated risk of risk gambling was observed among boys and individuals who did not disclose their gender, compared with girls. Additionally, students without a parent who had a university education were also more likely to engage in risk gambling compared with students with at least one university-educated parent.

**Fig. 1:**
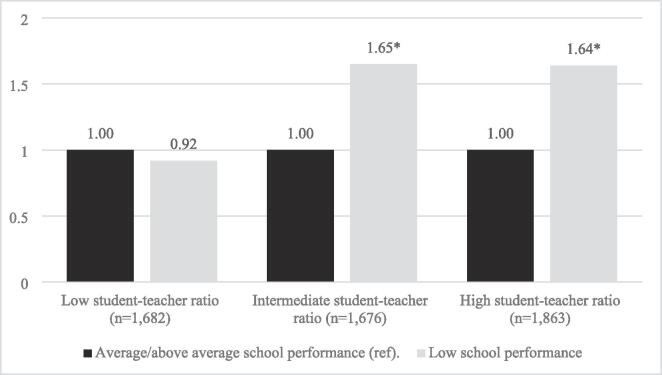
Student gambling by school performance and school student–teacher ratio. *Note*. Odds ratios from two-level binary logistic regressions controlling for school performance, gender, family structure, parental university education, migration background, the proportion of parents with post-secondary education, and the proportion of students with a foreign background. *p < 0.05.

**Fig. 2:**
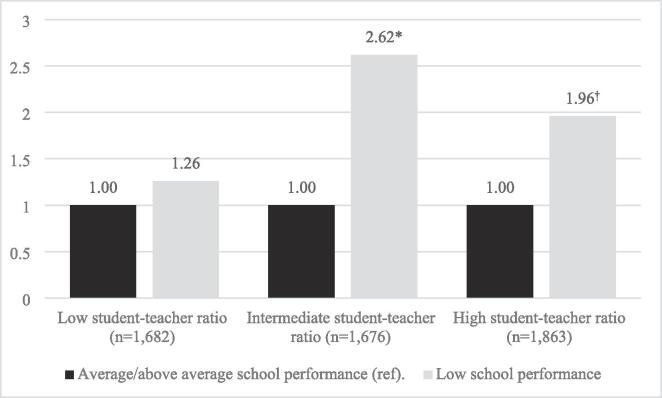
Student risk gambling by school performance and school student–teacher ratio. *Note*. *Odds ratios from two-level binary logistic regressions controlling for* school performance, gender, family structure, parental university education, migration background, the proportion of parents with post-secondary education, and the proportion of students with a foreign background. *p < 0.05 ^†^*p* < 0.10.

Next, we performed fully adjusted Models stratified by student–teacher ratio (using the same trichotomised variable as earlier). Results are presented in Fig. 1, Fig. 2, for gambling and risk gambling, respectively. As clearly illustrated by the figures, the association between low school performance and the likelihood of (risk) gambling was more pronounced in schools with an intermediate or high student–teacher ratio. The associations were strongest for schools with an intermediate student–teacher ratio, suggesting a threshold rather than a gradient pattern. Overall, the associations were similar for gambling and risk gambling, but were somewhat stronger in relation to risk gambling.

## 4 Discussion

The objective of this study was to investigate the moderating effect of student–teacher ratio on the association between school performance and youth gambling and risk gambling. Both direct and buffering effects were explored. We found evidence suggesting that schools’ teacher density may be critical for low performing students’ risk of engaging in gambling and risk gambling. The direct links between student–teacher ratio and (risk) gambling were however negligible. Put differently, this indicates that for students with average or above average grades, the student–teacher ratio makes little difference for their inclination to engage in gambling or risk gambling. By contrast, students who perform poorly in school seem to be protected from involving in gambling and, in particular, risk gambling by a higher teacher density. Having additional teachers in the classroom may provide targeted support to students who are facing challenges with their schoolwork. Consequently, this group of students could potentially derive greater advantages from a lower student–teacher ratio compared with their more academically successful peers. Furthermore, the findings of the present study align with previous research, indicating an elevated vulnerability to gambling among students who struggle academically, males, and individuals from lower socioeconomic backgrounds (Calado et al., 2017, Dowling et al., 2017, Svensson and Sundqvist, 2019).

While it is difficult to draw conclusions about mechanisms or causal effects based on the current study’s results, we can turn to the previous literature for possible interpretations. Several prior studies suggest that positive teacher-student relationships characterised by high levels of teacher support and teacher caring can protect youth from engaging in gambling (Elgar et al., 2018, Räsänen et al., 2016, Wahlström et al., 2022). In the case of teachers, it is reasonable to assume that having responsibility over fewer students may in greater capacity allow teachers to provide extra support for students who struggle in school. Stronger support from teachers could strengthen these students’ self-efficacy, study motivation and consequently also their prospects for the future, which have been inversely linked to gambling in prior studies (Bozzato et al., 2020, Låftman et al., 2020a). Higher teacher density could also facilitate stronger teacher-student bonds as well as improved relationships between teachers and parents (Blatchford et al., 2011, Ramberg et al., 2020, Rodriguez and Elbaum, 2014). Additionally, smaller class sizes have in previous research been linked to increased parental involvement and interest in school (Achilles & Finn, 2002). For students, experiencing strong bonds with teachers at school and having parents that take an active role in their education could be important factors for managing school (Barger et al., 2019, Roorda et al., 2017) and as such protect them from getting off track and involving in risky behaviours. Relatedly, students who perceive their relationships with teachers to be important and meaningful might be less inclined to participate in activities that they believe could disappoint their teachers and therefore avoid engaging in gambling (Chen and Feeley, 2018, Rudasill et al., 2010). A lower student–teacher ratio may also allow for better collaboration between teachers and an overall better learning environment (Cornelius et al., 2008, Jensen and Solheim, 2020) which, in line with research on effective schools, is central for student performance, well-being and behaviours (Alm et al., 2019, Bonell et al., 2013, Granvik Saminathen et al., 2018, Mortimore, 1993, Velasquez et al., 2013). One can moreover speculate that higher teacher density may be beneficial for the regulation of norms and for the school’s level of trust and informal control, elements that in previous research been linked to other risk behaviours (Olsson et al., 2017, Olsson et al., 2021a, Ramberg et al., 2019).

The results of the current study can have implications for various stakeholders concerned about student gambling. These include teachers, parents, and others who strive to address health-risk behaviours among adolescents. They can put forth the argument that a higher teacher density in schools not only improves the working conditions for school personnel but also has potential benefits for students both inside and outside the classroom. Moreover, schools with a lower student–teacher ratio could become more appealing to students if they are aware of the potential positive outcomes associated with stronger bonds formed with their teachers. By emphasizing the advantages of such connections, students and parents may be more inclined to choose schools with a lower student–teacher ratio. However, both in Sweden and elsewhere, there is already an ongoing discussion regarding the shortage of qualified teachers in school (See and Gorard, 2020, Swedish Schools Inspectorate, 2018). The high levels of stress experienced by teachers contribute significantly to this problem, frequently leading to premature exits from the profession (Casely-Hayford et al., 2022, Statistics Sweden, 2017). Although this study's findings suggest that increasing the number of teachers per student in upper secondary schools may serve as a safeguard against health-risk behaviours such as gambling, employing more teachers is a complex political issue that needs to be approached holistically (See & Gorard, 2020). Thus, beyond continuing the research on the link between teacher-related factors and student gambling, a realistic way to move forward is for policymakers to consider the potential positive effect on students' health-related behaviours that a higher teacher density can bring when discussing these issues.

### 4.1 Strengths and limitations

The current study benefits from using a large data material with linked school-level register data, allowing for reliable measures of schools’ student–teacher ratio. The questions that captured gambling and risk gambling have been used in prior studies and we could also control for relevant confounders. However, there are several limitations that should be considered. Firstly, since the data are cross-sectional, directions of the associations found are difficult to determine. Although poor academic performance is an established risk factor for gambling, engaging in risk behaviours could also affect school achievement negatively (Busch et al., 2014). Secondly, while we attempted to control for the selection of students into schools, there might still exist unobserved factors that are associated with both the likelihood of a student attending a particular school and their inclination to gamble. Lastly, since the data were collected from students in upper secondary schools in Stockholm only, the generalisability of the results to other age groups and geographical contexts is limited.

### 4.2 Conclusion

This study investigated the relationship between below-average school performance and gambling behaviour in a sample of 5,221 upper secondary students in Stockholm. We found that this association was less pronounced in schools with a lower student–teacher ratio (i.e., a higher teacher density). One advantage of focusing on factors within the school context is that they are comparatively more modifiable than individual and family-level factors. Based on our findings, increasing the number of teachers per student could prove to be a valuable investment, especially for students at risk of engaging in gambling. This consideration should be taken into account by policymakers concerned with addressing adolescent gambling. Moreover, it is reasonable to assume that a lower student–teacher ratio could have positive spillover effects on other outcomes and areas, benefiting the school and its student body as a whole. Additionally, it has the potential to contribute to a more sustainable and less stressful work environment for teachers.

### CRediT authorship contribution statement

**Joakim Wahlström:** Methodology, Formal analysis, Writing – original draft, Writing – review & editing, Funding acquisition. **Gabriella Olsson:** Conceptualization, Methodology, Formal analysis, Writing – original draft, Writing – review & editing, Funding acquisition.

## Declaration of Competing Interest

The authors declare that they have no known competing financial interests or personal relationships that could have appeared to influence the work reported in this paper.

## Data Availability

Data will be made available on request.
